# Molecular mechanisms of polychlorinated biphenyls in breast cancer: insights from network toxicology and molecular docking approaches

**DOI:** 10.3389/fphar.2025.1604993

**Published:** 2025-06-13

**Authors:** Xiaoyu Yang, Wenlong Liang, Zhenchu Feng, Guangyan Li, Xi Chen, Jianguo Zhang

**Affiliations:** Department of Mammary Surgery, The Second Affiliated Hospital of Harbin Medical University, Harbin, Heilongjiang, China

**Keywords:** polychlorinated biphenyls, breast cancer, immune cell infiltration, TCGA, molecular docking, network toxicology

## Abstract

**Background:**

Polychlorinated biphenyls (PCBs) are environmental pollutants associated with various health issues, including breast cancer. This study investigates potential molecular mechanisms by which PCBs may influence breast cancer progression using computational and preliminary experimental approaches.

**Methods:**

We conducted a differential expression analysis using the TCGA-BRCA dataset. PCBs-related toxicological targets were collected from the Comparative Toxicogenomics Database (CTD). Enrichment and pathway analyses identified candidate biological processes and pathways. Protein-protein interaction (PPI) networks were constructed to identify hub genes. Single-cell expression levels of key targets were analyzed (GSE114727 dataset). Molecular docking predicted binding affinities of PCBs congeners with key targets. Cell experiments assessed gene expression changes upon PCBs exposure.

**Results:**

We identified 52 upregulated and 24 downregulated PCBs-related toxicological targets in breast cancer. Enrichment analysis highlighted potential associations with pathways such as PI3K-Akt, MAPK, and HIF-1, including genes like BRCA1, FGFR1, IGF1, AKT1, and EGF. PPI network analysis identified key hub genes like EZH2, EGF, BRCA1, AKT1, IL6, and TNF. Single-cell analysis suggested variable expression of key targets across immune cell types. Molecular docking predicted strong binding affinities of PCB 105 with EZH2 and EGF *in silico*. Pathway analysis indicated gene expression alterations in the PI3K-AKT and MAPK signaling upon PCBs exposure, though causal relationships remain to be validated.

**Conclusion:**

Our integrated analysis proposes that PCBs exposure may perturb key molecular pathways in breast cancer. Computational findings implicate targets like EZH2 and EGF, while preliminary cell experiments support further investigation. These results highlight a need for mechanistic studies to confirm PCB-induced effects and their therapeutic relevance, underscoring environmental pollutants as potential risk factors in cancer.

## 1 Introduction

Polychlorinated biphenyls (PCBs) are a group of synthetic organic chemicals extensively used in industrial applications due to their chemical stability and insulating properties (Zarerad et al., 2023). Although many countries have banned PCBs, their persistence in the environment means they continue to be detected in various ecosystems (Liu et al., 2020). Recent studies show that PCBs remain a significant concern due to their long-lasting presence in the environment, particularly in sediments, water bodies, and wildlife, despite regulatory bans (Beyer and Biziuk, 2009). The environmental impact of PCBs is profound, as they not only accumulate in the food chain but also disrupt local ecosystems, affecting biodiversity and ecological balance. This environmental persistence poses significant health risks. Epidemiological studies and experimental data have demonstrated a link between PCBs exposure and an increased risk of several cancers, including breast cancer (Pourhassan et al., 2022; Zani et al., 2013). This association is largely attributed to PCBs’ endocrine-disrupting properties, which can interfere with hormonal regulation and contribute to carcinogenesis (Chen et al., 2015). Additionally, ongoing exposure to PCBs continues to occur through contaminated food sources (e.g., fish and dairy), occupational settings, and legacy contamination in urban areas and older buildings (Ritter et al., 2002; Osemwengie and Morgan, 2019; Malisch and Kotz, 2014). Understanding the environmental pathways through which PCBs enter and affect human health is crucial for developing effective mitigation strategies. Investigating the molecular mechanisms through which PCBs influence breast cancer progression is crucial for understanding their carcinogenic potential and for developing targeted interventions to mitigate their impact.

Breast cancer, one of the predominant neoplasms affecting women worldwide, is shaped by a multifaceted interaction of environmental exposures, lifestyle choices, and genetic predispositions (Ba et al., 2020). The escalating prevalence of breast cancer has been partially linked to environmental determinants, with prolonged exposure to chemical agents, such as PCBs, being scrutinized for their potential contributory effects (Parada et al., 2016). Despite the regulatory bans, exposure to PCBs is still prevalent, and the toxicity of these pollutants continues to affect public health, particularly in regions with legacy contamination or in populations with higher levels of exposure due to diet or occupation. Distinct functional PCB congeners exhibit varying correlations with prognostic biomarkers of breast carcinoma, as well as with the classification stages of tumors (Qiu et al., 2020). Furthermore, PCBs may elevate the risk of short-term breast cancer-specific mortality and long-term all-cause mortality in breast cancer patients (Parada et al., 2020). Previous research has demonstrated that PCBs can disrupt hormone regulation and activate cancer-related signaling pathways (Guo et al., 2020; Qin et al., 2021). Recent studies have highlighted that five specific PCB congeners (PCB 99, PCB 105, PCB 118, PCB 138, and PCB 183) are associated with an increased risk of breast cancer, establishing them as critical subjects for investigation (Liu et al., 2023). However, the evidence linking these PCBs to breast cancer remains limited and sometimes contradictory, and epidemiological studies on this topic have produced varying results. In addition, a recent meta-analysis suggests that PCB 99, PCB 183, and PCB 187 specifically elevate the risk of developing breast cancer (Leng et al., 2016). These congeners were selected for our study due to their documented presence in environmental samples and their potential association with breast cancer, as indicated by a few studies. However, the specific molecular mechanisms and pathways modulated by these PCBs in the context of breast cancer remain poorly understood and need further investigation. Studies have predominantly focused on general toxicological effects, with insufficient emphasis on comprehensive molecular interaction networks and pathways at a systems biology level. Additionally, data on the binding affinities and specific interactions of PCBs congeners with key protein targets in breast cancer are sparse. This gap necessitates a more integrative and detailed analysis to elucidate the intricate molecular interplay induced by PCBs exposure.

Understanding how PCBs influence breast cancer at the molecular level is critical for several reasons. First, it provides deeper insights into the carcinogenic mechanisms of persistent environmental pollutants. Second, it helps identify potential biomarkers for early detection and risk assessment of breast cancer related to PCBs exposure. Lastly, it contributes to the development of targeted therapies and preventive strategies. Given the ongoing exposure to PCBs through various environmental and occupational sources, addressing these knowledge gaps remains of paramount importance for public health. This research seeks to clarify the molecular mechanisms by employing network toxicology and molecular docking approaches. The focus is on examining the interactions between PCBs and key proteins that play a significant role in the progression of breast cancer.

## 2 Methods

### 2.1 Collection of PCB targets from the CTD

CTD (http://ctdbase.org/, accessed October 2024) is a comprehensive resource that contains various data regarding chemical exposures and their biological effects. This includes information on chemicals, genomics, phenotypes, pathologies, and taxonomies sourced from scientific literature (Davis et al., 2023). First, we used the CTD’s built-in ‘Analyze’ tool with the default parameters to identify genes associated with the six PCBs (PCB 99, PCB 105, PCB 118, PCB 138, PCB 183, and PCB 187) by searching the ‘Chemicals’ module using CAS registry numbers. We then applied the ‘Set Analyzer’ tool to cross-reference these genes with breast cancer-associated genes. The query specifications included: (1) selecting ‘Enriched Diseases’ and ‘Breast Neoplasms’, and (2) applying a p-value threshold of <0.01.

### 2.2 Identifying genes linked to breast cancer by analyzing TCGA data

We obtained the breast cancer-related dataset from the TCGA database (https://portal.gdc.cancer.gov). This dataset comprises 113 normal breast samples and 1,113 breast cancer samples. We performed differential expression gene analysis by applying the criteria of |log fold change (FC)| ≥ 1 and an adjusted p-value <0.05 to filter the differentially expressed genes (DEGs). Using the Venny tool, we identified the overlap between breast cancer-related targets and those associated with PCBs. This intersection highlights potential targets that may explain the toxic effects of PCBs in triggering breast cancer. The results are shown using volcano and heatmap visualizations created with the ggplot2 and ComplexHeatmap packages.

### 2.3 Enrichment analysis

To elucidate the essential molecular functions, biological mechanisms, and pathways that may play a role in the onset of breast cancer, particularly about exposure to PCBs, we carried out analyses of Gene Ontology (GO) functional enrichment and Kyoto Encyclopedia of Genes and Genomes (KEGG) pathways. The enrichment evaluation was executed using the ClusterProfiler package, with a significance threshold set at p < 0.05 to pinpoint relevant biological pathways. The resulting analytical data were subsequently visualized through the use of the ggplot2 package.

### 2.4 Identification of hub toxicological targets of PCBs

A PPI network focusing on the toxicological targets of PCBs was constructed using data from the STRING database (https://cn.string-db.org/), setting a confidence score threshold of 0.7 (Szklarczyk et al., 2023). Following this, the network was visualized using Cytoscape software. Subsequently, we employed the CytoHubba plugin to identify central targets utilizing three topological analysis methodologies: degree centrality, closeness centrality, and betweenness centrality (Bai et al., 2024). Degree centrality measures the number of connections a node has within the network, identifying genes that interact with the greatest number of other genes. Closeness centrality quantifies how close a node is to all other nodes in the network, highlighting genes that can potentially influence the entire network due to their central position. Betweenness centrality reflects how often a node acts as a bridge along the shortest paths between other nodes, identifying genes that control communication between different parts of the network. The top 10 genes were extracted for each methodology, and the overlapping genes were designated as the hub targets. These centrality measures were chosen because they collectively capture different aspects of network structure, helping to identify genes that are crucial in the context of PCBs toxicity and breast cancer. To quantitatively evaluate the differential expression of hub targets in breast cancer tissues relative to normal control tissues, RNA-seq data from the TCGA-BRCA cohort were utilized. Gene expression was visualized through violin plots generated via the R package ggplot2. The Wilcoxon rank-sum test was employed to determine the expression level disparities between these two tissue types.

### 2.5 Analysis of core targets and tumor immune correlation

The ESTIMATE and ssGSEA methodologies were employed for the analysis of the immune microenvironment. A correlation heatmap was constructed utilizing the pheatmap package. Spearman correlation analysis was conducted using the ggplot2 package.

### 2.6 Tumor immune single cell hub (TISCH)

The TISCH database (accessible at http://tisch.comp-genomics.org/) comprises a wide range of single-cell RNA sequencing datasets that provide essential insights into the complex characteristics of the tumor microenvironment (Han et al., 2023). In this study, we analyzed the expression profiles of key hub genes across various immune cell types utilizing the GSE114727 dataset obtained from TISCH.

### 2.7 Cell experiments

The MCF-7 cell line was sourced from the American Type Culture Collection. These cells were grown in RPMI-1640 medium, acquired from Life Technologies in Shanghai, China. The medium was enriched with 10% fetal bovine serum and 1% penicillin-streptomycin antibiotics. The cell cultures were kept in a controlled environment at 37°C with 5% CO_2_. For experimental purposes, the MCF-7 cells were exposed to either 1 μM PCBs or a DMSO vehicle control for 48 h. After this period, the cells were washed twice with phosphate-buffered saline (PBS) and then collected for further analysis. RNA was isolated from the cells using an RNA Extraction Reagent from Thermo Fisher Scientific. Following RNA extraction, cDNA was synthesized using the PrimeScript RT Reagent Kit. Gene expression analysis was conducted via quantitative reverse transcription polymerase chain reaction (RT-qPCR) on the ABI 7900HT PCR system by Applied Biosystems. The relative expression levels of genes were determined using the 2^−ΔΔCT^ method.

### 2.8 Molecular docking

The compound structures of PCBs were sourced from the PubChem repository (https://pubchem.ncbi.nlm.nih.gov/), specifically in SDF (Structure Data File) format. This initial data set underwent processing through AutoDock Tools (version 1.5.6). Following this processing, the files were converted into PDBQT format. To conduct the docking experiments, the structures of the target proteins were retrieved from the Protein Data Bank (PDB) (https://www.rcsb.org). The pre-processing of these protein structures was carried out using AutoDock Tools as well. This step involved several essential tasks, such as the removal of water molecules that could interfere with binding assessments, the addition of hydrogen atoms to reflect a more realistic molecular structure, and the assignment of appropriate charges to the molecules. The actual docking simulations were executed using AutoDock Vina, a powerful tool for predicting the binding affinity and elucidating the interaction patterns between the ligand, in this case the PCBs, and the specific target protein (Trott and Olson, 2010). To better understand and illustrate these ligand-protein interactions, the results were visualized with PyMOL software, version 1.0.0.

### 2.9 Statistical analysis

Results are presented as mean ± SD. Differences between the two groups were assessed using Student’s t-test, with statistical significance defined as p < 0.05.

## 3 Results

### 3.1 Network toxicology analysis

#### 3.1.1 Differential expression analysis of PCBs-related toxicological targets in breast cancer

**FIGURE 1:**
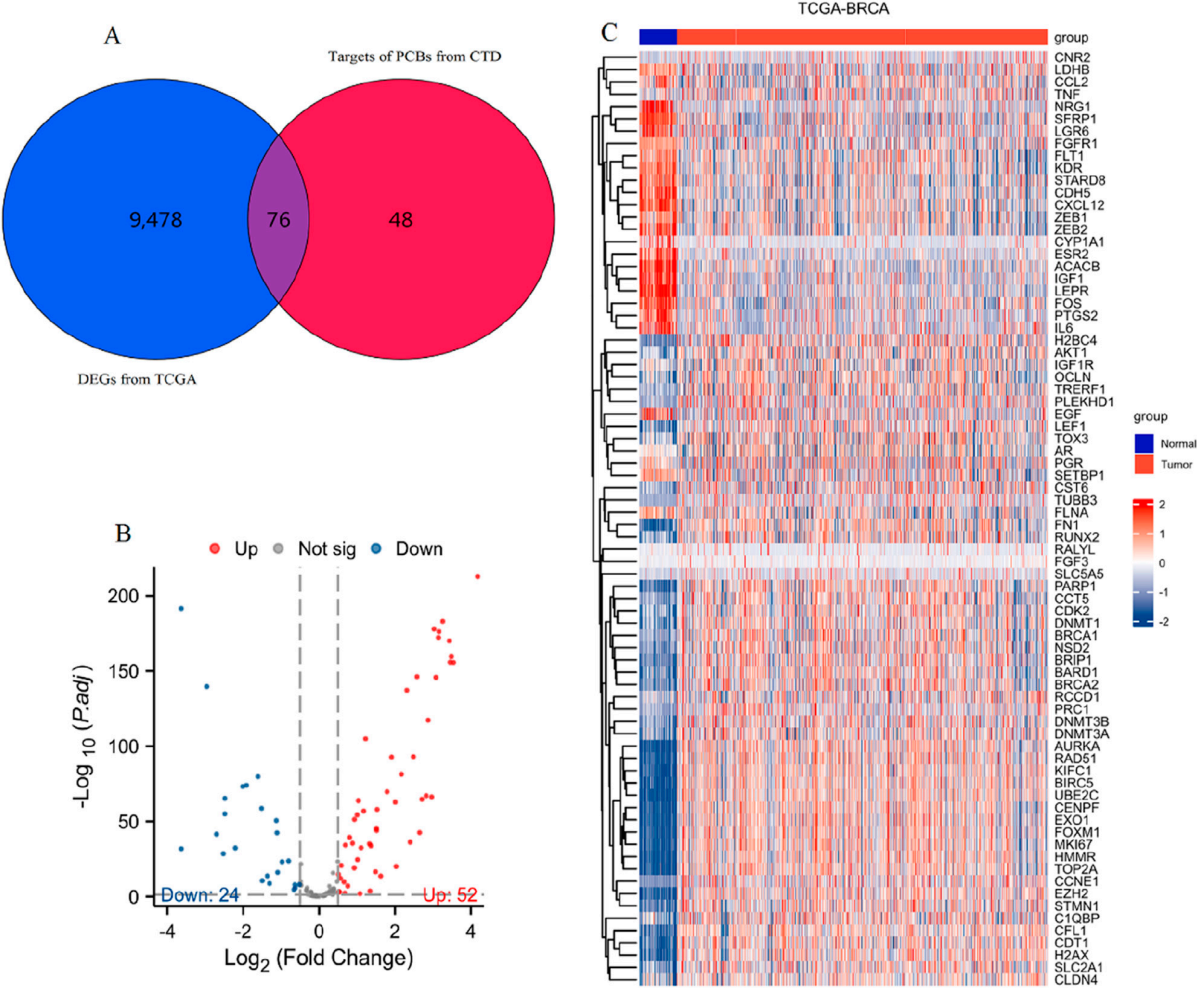
Identification and expression analysis of differentially expressed PCBs-related toxicological targets in breast cancer. **(A)** Venn diagram showing the intersection of DEGs from TCGA-BRCA (blue) and targets of PCBs from CTD (red). **(B)** Volcano plot visualizing the 76 differentially expressed PCBs-related targets. Red and blue dots indicate significantly upregulated and downregulated genes, respectively. **(C)** Heatmap illustrating the expression profiles of the 76 differentially expressed PCBs-related targets in normal (blue) and tumor (red) samples from the TCGA-BRCA dataset.

From 9554 DEGs (Supplementary Table S1) and 124 PCBs-related targets, 76 genes emerged as differentially expressed PCBs-related toxicological targets (Figure 1A). The visualization of these 76 targets using a volcano plot is shown in Figure 1B. Specifically, 52 genes are upregulated while 24 genes are downregulated. Figure 1C presents a heatmap detailing the expression profile of these 76 toxicological targets across the TCGA-BRCA dataset. This visualization highlights the distinct expression patterns of these gene targets between normal and tumor tissues.

#### 3.1.2 Enrichment analysis of PCBs-related toxicological targets

**FIGURE 2:**
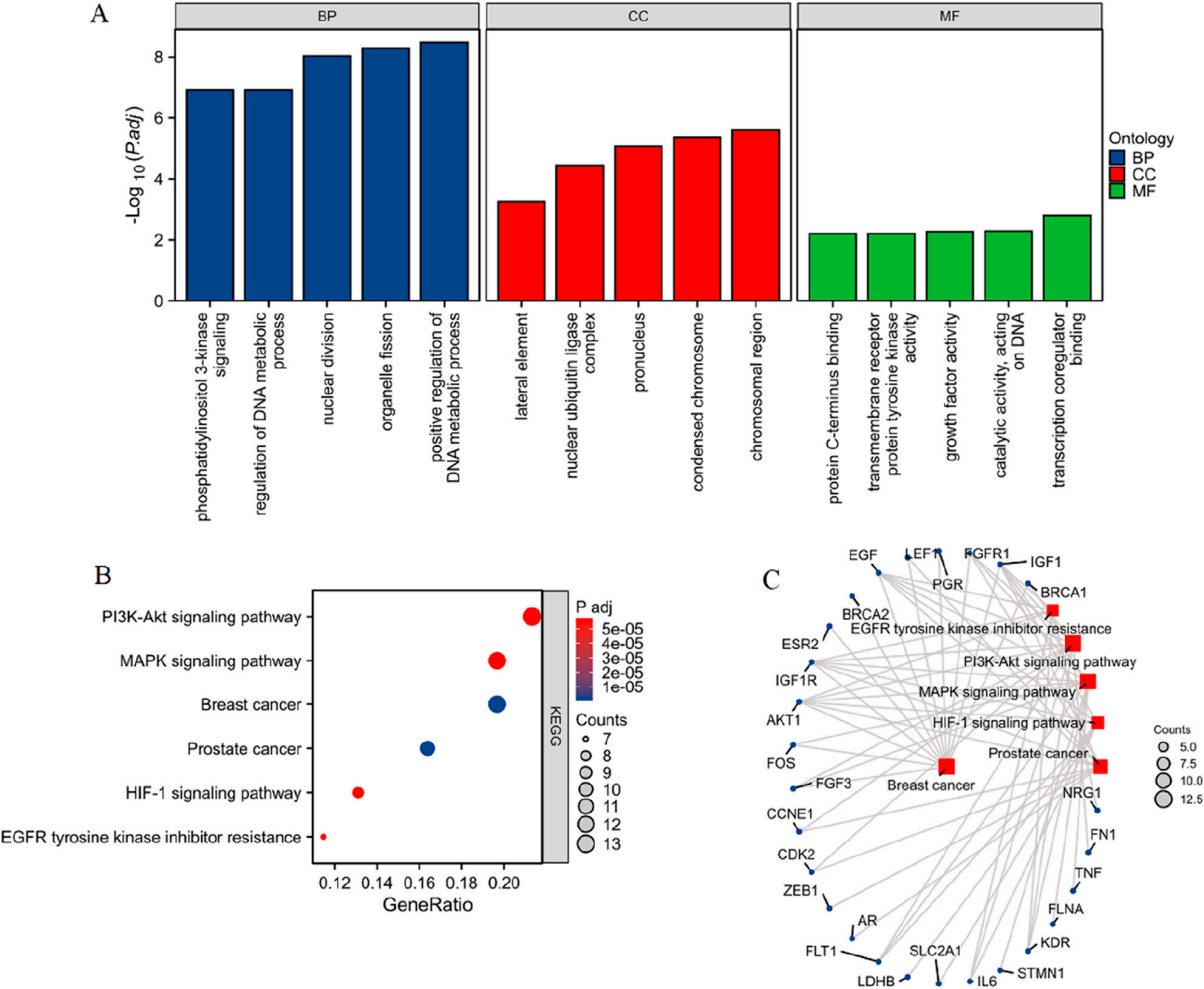
Enrichment analysis of PCBs-related toxicological targets in breast cancer. **(A)** GO enrichment analysis of the 76 differentially expressed PCBs-related targets. The bar plots display significantly enriched terms across three categories: BP in blue, CC in red, and MF in green. **(B)** KEGG pathway enrichment analysis illustrating the significantly enriched pathways. The x-axis indicates the gene ratio, and the dot size corresponds to the number of genes involved, while the color gradient represents the adjusted p-value. **(C)** Network visualization of the enriched pathways and their associated genes. The node size represents the number of genes involved in each pathway.

The 76 differentially expressed PCBs-related toxicological targets were further subjected to enrichment analysis, the results of which are summarized in Figure 2 and Supplementary Table S2. Figure 2A represents the Gene Ontology (GO) enrichment analysis across three categories: BP (blue), CC (red), and MF (green). Key enriched BP terms include phosphatidylinositol 3-kinase signaling, regulation of DNA metabolic process, nuclear division, organelle fission, and positive regulation of DNA metabolic process. For CC, critical terms include the lateral element, nuclear ubiquitin ligase complex, pronucleus, and condensed chromosome. Similarly, MF enrichment analysis indicates significant terms such as protein C-terminus binding, ATPase activity, transcription coregulator binding, and catalytic activity acting on DNA. Figure 2B showcases the KEGG pathway enrichment analysis of the 76 toxicological targets. Notably enriched pathways include PI3K-Akt signaling pathway, MAPK signaling pathway, breast cancer, prostate cancer, HIF-1 signaling pathway, and EGFR tyrosine kinase inhibitor resistance. Figure 2C provides a network visualization of the KEGG pathways and their associated DEGs. Key pathways such as PI3K-Akt signaling, MAPK signaling, and breast cancer are linked with multiple targets including BRCA1, FGFR1, IGF1, AKT1, and EGF, suggesting their potential involvement in breast cancer pathology influenced by PCBs exposure.

#### 3.1.3 PPI network analysis reveals key regulatory genes among PCBs-related targets

**FIGURE 3:**
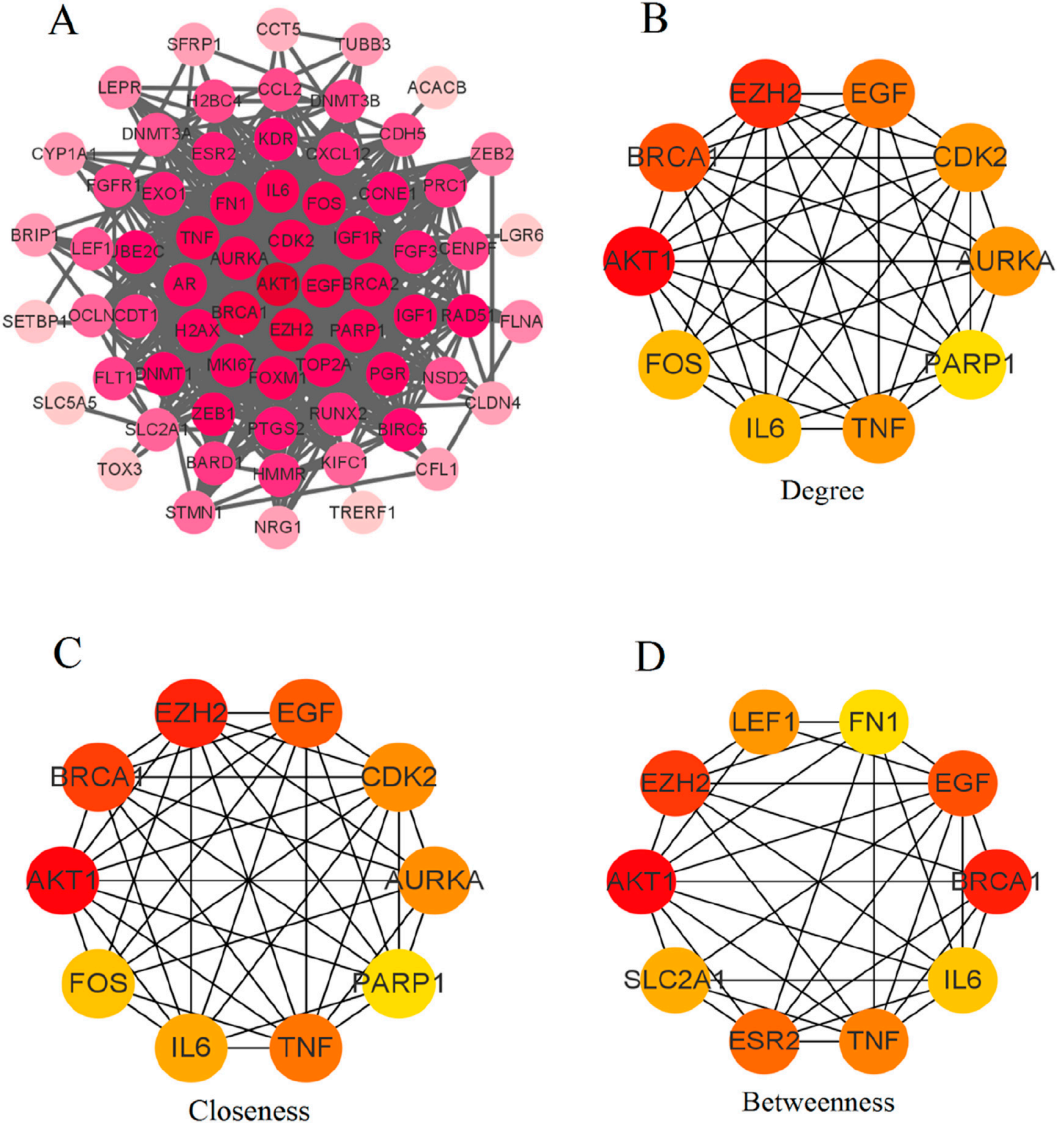
PPI network analysis of differentially expressed PCBs-related toxicological targets in breast cancer. **(A)** Comprehensive PPI network of the 76 differentially expressed genes. Nodes represent individual genes, and edges represent predicted interactions between them. **(B)** Degree centrality analysis identifies the top 10 hub genes based on their number of direct interactions. **(C)** Closeness centrality analysis ranks the top 10 hub genes based on their proximity to all other nodes in the network. **(D)** Betweenness centrality analysis lists the hub genes that act as critical intermediaries within the network.

Figure 3A displays the comprehensive PPI network of the 76 differentially expressed genes. Nodes represent individual genes, while edges represent the predicted interactions between them. The network reveals a dense core of interconnected nodes, suggesting significant interaction among the majority of these targets. Figure 3B shows the top 10 hub genes in the network based on degree centrality. Key hub genes identified include EZH2, EGF, CDK2, BRCA1, AKT1, IL6, TNF, PARP1, FOS, and AURKA. Figure 3C ranks the top hub genes by closeness centrality. Similar key genes are identified. Figure 3D lists the hub genes according to betweenness centrality, which identifies genes (LEF1, FN1, EGF, BRCA1, IL6, TNF, ESR2, SLC2A1, EZH2, and AKT1) that act as critical intermediaries in the network.

#### 3.1.4 Identification and expression analysis of key toxicological targets in breast cancer

**FIGURE 4:**
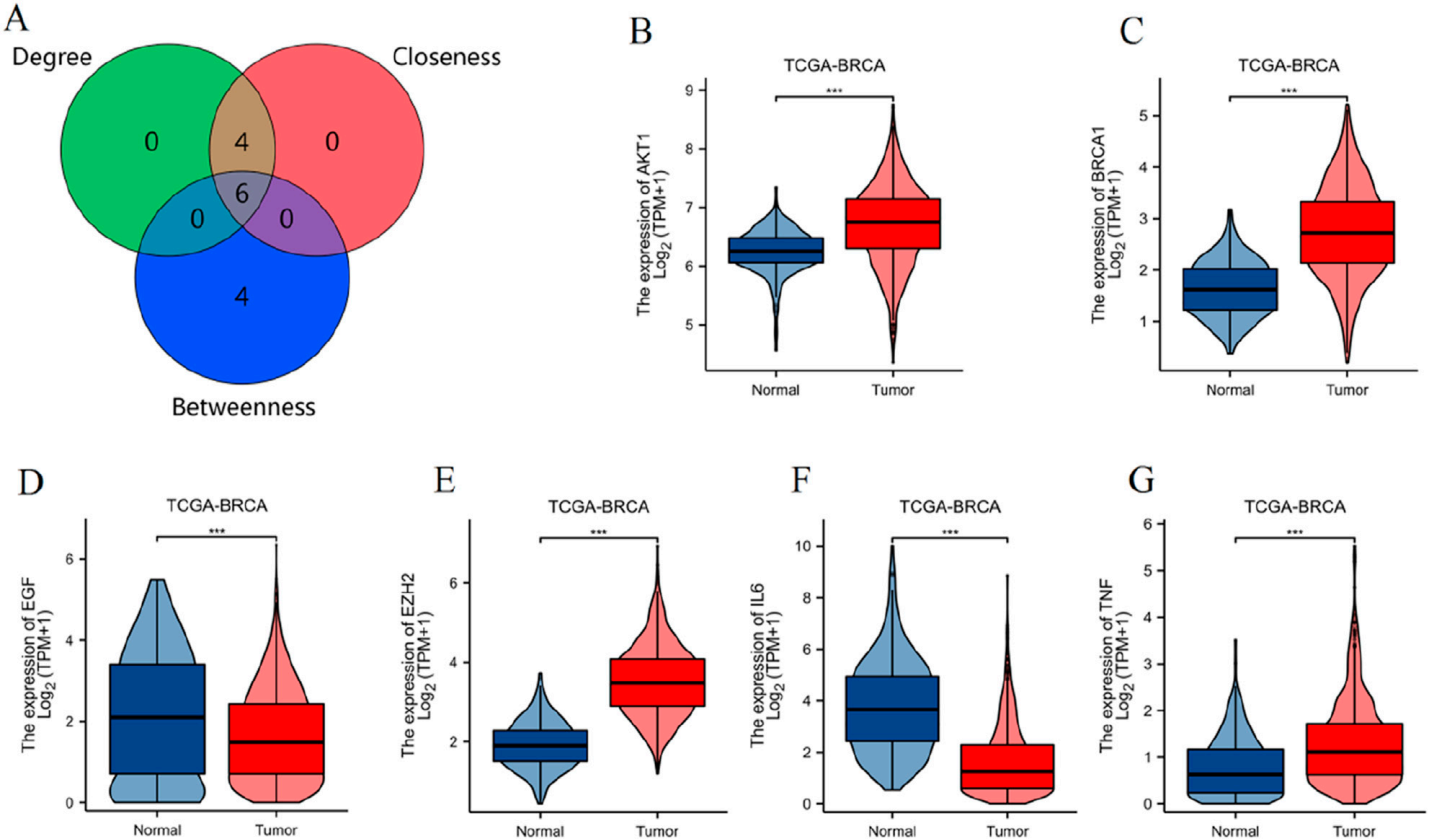
Identification and expression analysis of key toxicological targets of PCBs. **(A)** Venn diagram illustrating the intersection of key targets identified using three CytoHubba topological analysis algorithms: Degree, Closeness, and Betweenness. Violin plots displaying the expression levels of the six key targets in the TCGA-BRCA dataset, comparing normal and tumor tissue samples. The targets include: **(B)** AKT1, **(C)** BRCA1, **(D)** EGF, **(E)** EZH2, **(F)** IL6, **(G)** TNF. ***p < 0.001.

Using the CytoHubba plugin with three topological analysis algorithms (Degree, Closeness, and Betweenness), we identified 6 key targets from 76 differentially expressed PCBs-related toxicological targets, as visualized in the Venn diagram (Figure 4A). Subsequently, we analyzed the gene expression levels of these 6 core targets using the TCGA-BRCA dataset (Figures 4B–G). The results demonstrate significant differential expression between normal and tumor tissues for each of the targets.

#### 3.1.5 Differential expression of key PCBs-related toxicity targets in breast cancer subtypes

As shown in Supplementary Figure S1, AKT1, BRCA1, and EGF expressions exhibited a marked decrease in ER-negative tumors (p < 0.001). EZH2, IL6, and TNF expressions were all significantly elevated in ER-negative tissues compared to ER-positive ones, with statistical significance maintained across all targets (p < 0.001). Supplementary Figure S2 illustrates the comparisons of these targets between HER2-negative and HER2-positive breast cancer samples. The expression of AKT1 showed a significant increase in HER2-positive samples compared to HER2-negative samples (p < 0.01). IL6 expression was significantly lower in HER2-positive samples than in HER2-negative samples (p < 0.01). The expression levels of BRCA1, EGF, EZH2, and TNF did not show significant differences between HER2-negative and HER2-positive subtypes. As illustrated in Supplementary Figure S3, expressions of AKT1, BRCA1, and EGF demonstrated a pronounced reduction in PR-negative tumors (p < 0.001). Conversely, expressions of EZH2, IL6, and TNF were all considerably heightened in PR-negative tissues in comparison to ER-positive ones (p < 0.01).

#### 3.1.6 Correlation analysis between key PCBs-related toxicity targets and tumor immune microenvironment

**FIGURE 5:**
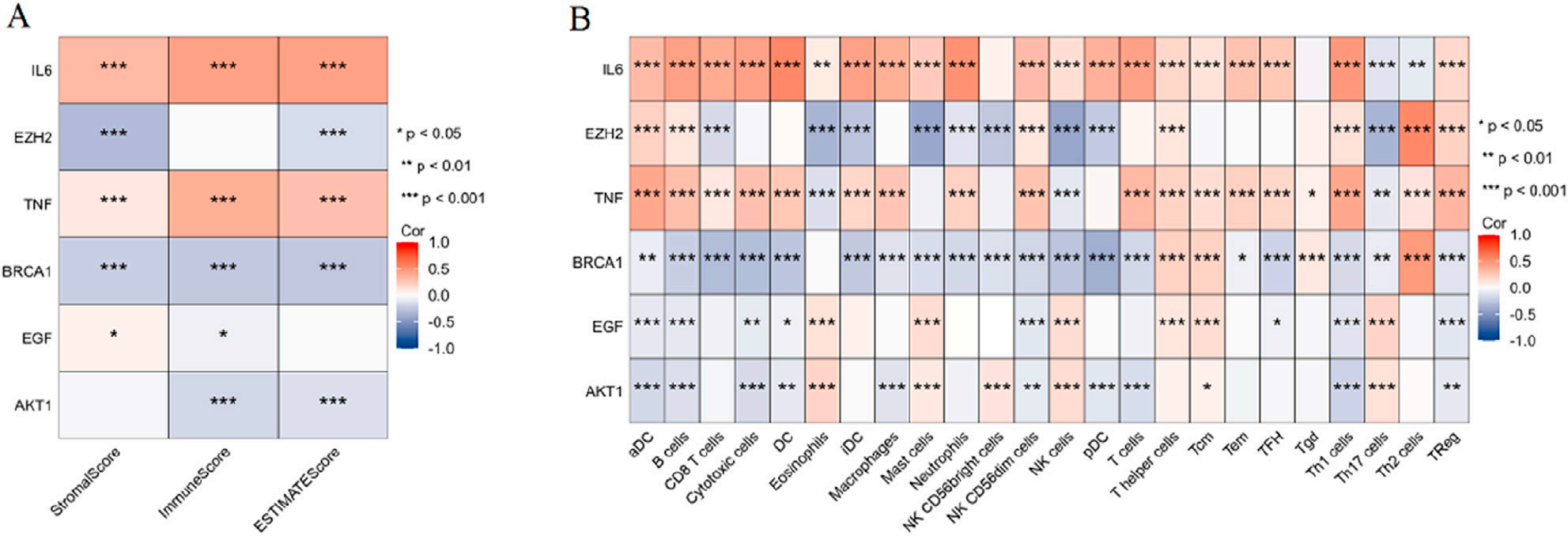
Correlation analysis between key PCBs-related toxicity targets and tumor immune microenvironment. **(A)** Heatmap depicting the correlation between six key PCBs-related toxicity targets and the ESTIMATE scores (StromalScore, ImmuneScore, and ESTIMATEScore). **(B)** Heatmap illustrating the correlation between the same six PCBs-related toxicity targets and ssGSEA-derived immune cell infiltration scores across various immune cell types. Red denotes positive correlations, blue denotes negative correlations. Significance levels are indicated as follows: *p < 0.05, **p < 0.01, and ***p < 0.001.

As shown in Figure 5A, the ESTIMATE scores, StromalScore, and ImmuneScore were analyzed for their correlation with the expression of six key genes. Notably, IL6 and TNF showed a significant positive correlation with all three scores (p < 0.001). EZH2 exhibited significant negative correlations with StromalScore and ESTIMATEScore (p < 0.001). BRCA1 exhibited significant negative correlations with all three scores (p < 0.001). AKT1 exhibited significant negative correlations with ImmuneScore and ESTIMATEScore (p < 0.001). As shown in Figure 5B, the correlation analysis extended to ssGSEA-derived immune cell infiltration scores for various immune cells. Important findings include: IL6 and TNF displayed significant positive correlations across multiple immune cell types, such as aDC, B cells, DC, macrophages, T cells, CD8 T cells, and Treg cells (p < 0.001). Contrarily, EZH2 and BRCA1 revealed significant negative correlations with several immune cell types, notably CD8 T cells, mast cells, NK cells, and Th17 cells (p < 0.001). EGF and AKT1 revealed significant negative correlations with several immune cell types, notably aDC, B cells, and Th1 cells (p < 0.001).

#### 3.1.7 Expression levels of key PCBs-related toxicity targets in tumor immune cells

**FIGURE 6:**
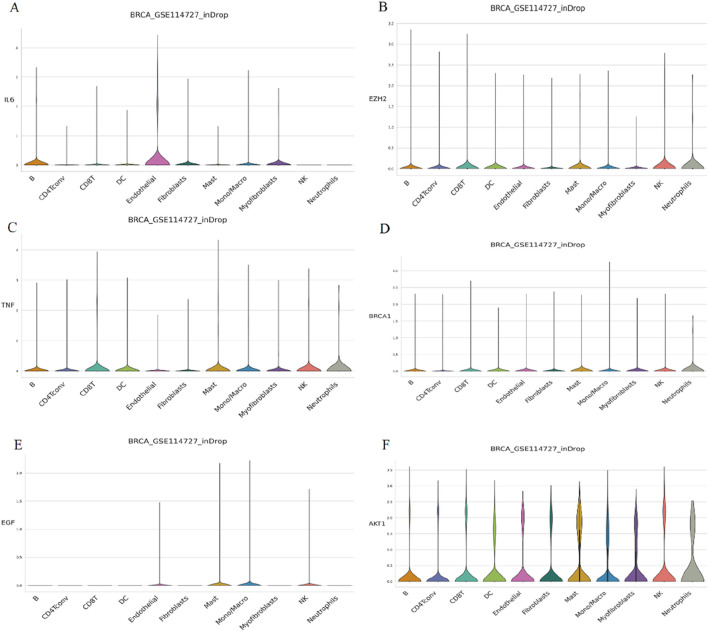
Single-cell expression analysis of key PCBs-related toxicity targets in immune cells. The targets assessed are **(A)** IL6, **(B)** EZH2, **(C)** TNF, **(D)** BRCA1, **(E)** EGF, and **(F)** AKT1. The height and width of each violin represent the distribution and frequency of gene expression within each cell type.

To assess the expression abundance of six key PCBs-related toxicity targets at the single-cell level, we analyzed the breast cancer dataset GSE114727. The violin plot demonstrates that IL6 expression is highly enriched in endothelial cells and B cells, with minimal expression observed in other immune cell types (Figure 6A). EZH2 expression is relatively low across all immune cell types. However, a slight increase in expression is noted in neutrophils and NK cells (Figure 6B). The expression of TNF is distributed across a range of immune cell types, with a modest increase observed particularly in neutrophils, NK cells, mast cells, and CD8 T cells (Figure 6C). BRCA1 shows low expression levels across all immune cell subsets evaluated. A slightly higher expression is recorded in mast cells and neutrophils (Figure 6D). EGF expression levels are minimal in all immune cell types (Figure 6E). AKT1 is expressed across a broad range of immune cell types, with higher expression levels in mast cells and neutrophils (Figure 6F).

#### 3.1.8 Representative molecular pathways of breast cancer induction by PCBs congeners exposure

**FIGURE 7:**
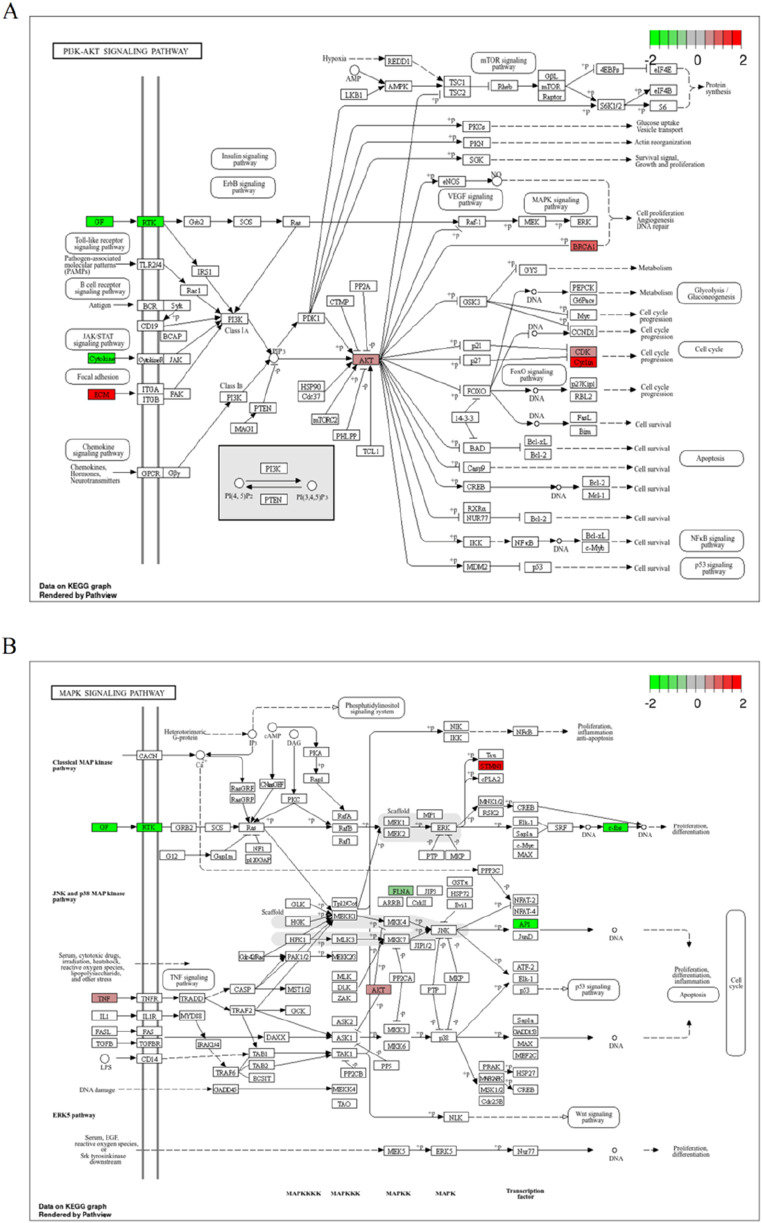
Representative molecular pathways of breast cancer induction by PCBs compound exposure. **(A)** Illustration of the PI3K-AKT signaling pathway displaying gene expression changes upon PCBs exposure. **(B)** Depiction of the MAPK signaling pathway with gene expression alterations due to PCBs exposure. Upregulated genes are marked in red, and downregulated genes in green.

**FIGURE 8:**
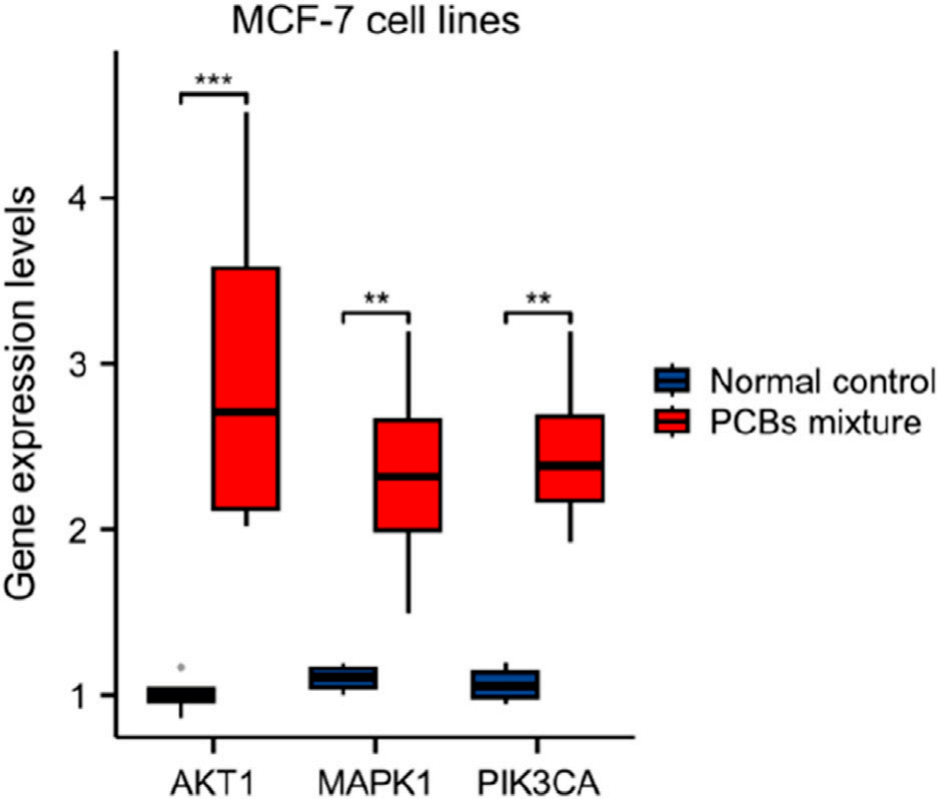
Gene expression levels of AKT1, MAPK1, and PIK3CA in MCF-7 cell lines following exposure to PCBs mixture. The box plot shows the expression levels of key genes involved in breast cancer pathways. The red boxes represent gene expression levels in cells exposed to the PCBs mixture, while the blue boxes represent the normal control group. Significance levels are indicated as follows: **p < 0.01, and ***p < 0.001.

As shown in Figure 7A, the PI3K-AKT signaling pathway was analyzed for changes in gene expression upon PCBs exposure. Significant alterations include: The heightened expression of AKT1, a key component of this signaling pathway, is likely linked to processes such as cell survival and the progression of the cell cycle. Similarly, the increased levels of BRCA1 play a significant role in DNA repair mechanisms, angiogenesis, and the proliferation of cells. The MAPK signaling pathway, integral to cell proliferation, differentiation, and apoptosis, showed significant perturbations in response to PCBs exposure (Figure 7B). In addition, the expression levels of pivotal genes in the representative molecular pathways implicated in breast cancer were analyzed following exposure to PCBs. In MCF-7 cell lines, a significant upregulation of AKT1, MAPK1, and PIK3CA gene expression was observed in the PCBs mixture group compared to the normal control (Figure 8). These results indicate a clear upregulation of these genes in response to PCBs exposure in MCF-7 cell lines, suggesting a potential mechanism through which PCBs contribute to breast cancer pathogenesis.

### 3.2 Molecular docking analysis

#### 3.2.1 Molecular docking analysis of PCBs congeners with key toxicity targets

**FIGURE 9:**
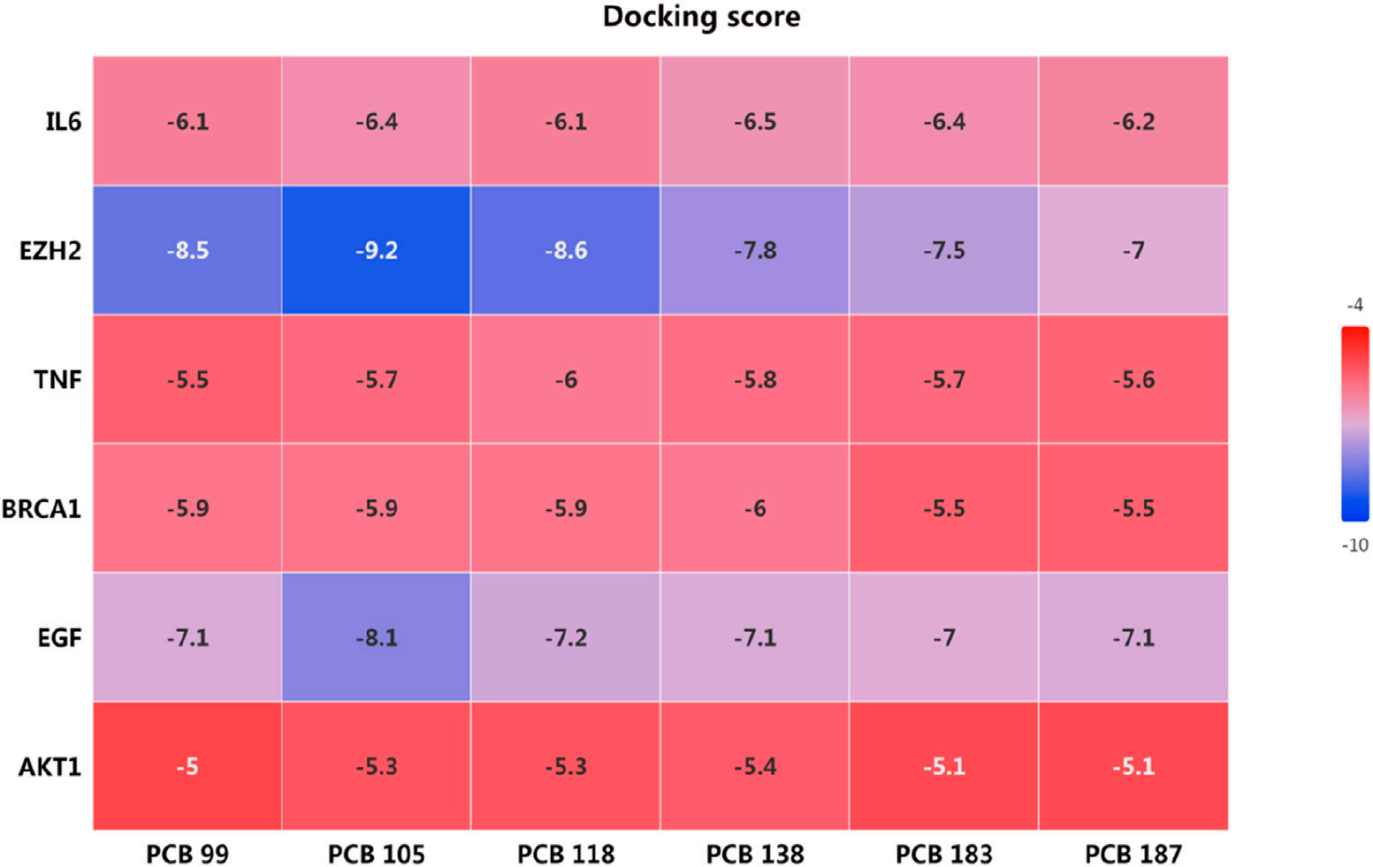
Molecular docking scores of PCBs congeners with key toxicity targets.

The docking scores, presented in the heatmap (Figure 9), indicate the binding affinities of each PCB compound to the respective targets. Lower docking scores signify stronger binding affinities. The docking scores for IL6 with the PCBs congeners ranged from −6.1 to −6.5, indicating moderate binding affinities. The lowest docking score was observed with PCB 138 (−6.5). EZH2 exhibited the lowest docking scores among all targets, with values ranging from −7 to −9.2. Particularly, PCB 105 showed the strongest binding affinity with EZH2 (−9.2), followed by PCB 118 (−8.6). The docking scores for TNF with PCBs congeners ranged from −5.5 to −6.0. The highest binding affinity was observed with PCB 118 (−6.0). The binding affinities of BRCA1 with PCBs congeners were relatively consistent, with docking scores between −5.5 and −6.0. The highest affinity was noted with PCB 138 (−6.0). The docking scores for EGF ranged from −7.0 to −8.1, showing strong binding affinities. PCB 105 exhibited the strongest interaction with EGF (−8.1). AKT1 had the highest docking scores amongst the targets, indicating weaker binding affinities with values ranging from −5.0 to −5.4. The lowest score was observed with PCB 138 (−5.4). Overall, the molecular docking analysis revealed that PCB 105 possesses the strongest binding affinities across several key targets, particularly EZH2 and EGF.

#### 3.2.2 Optimal docking poses of PCBs congeners with EZH2

**FIGURE 10:**
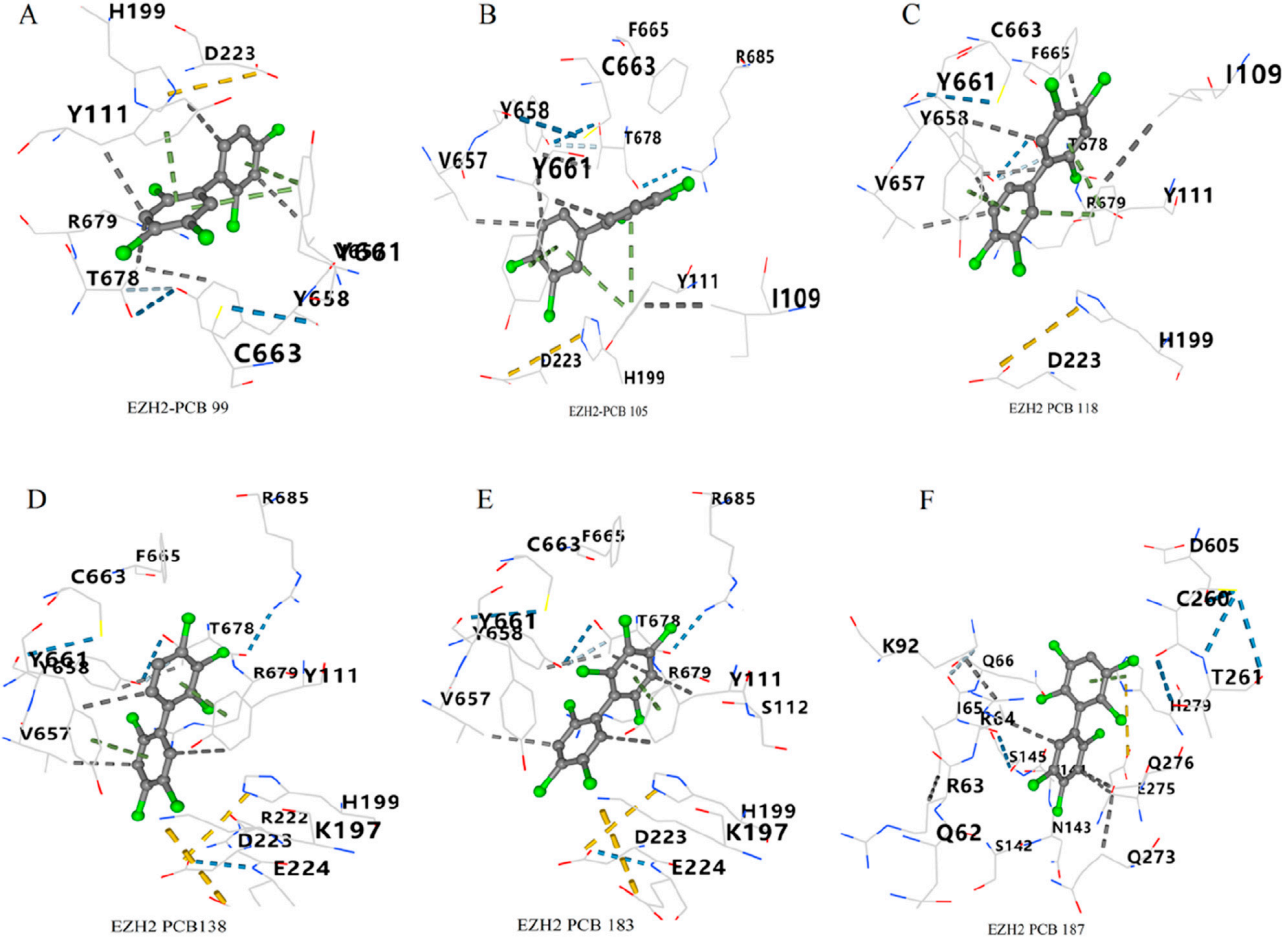
Optimal docking poses of PCBs congeners with EZH2. Molecular interactions between six PCBs congeners and the key toxicity target EZH2 are illustrated. The optimal docking poses are shown for **(A)** PCB 99, **(B)** PCB 105, **(C)** PCB 118, **(D)** PCB 138, **(E)** PCB 183, and **(F)** PCB 187.

As shown in Figure 10A, the docking pose of PCB 99 with EZH2 shows the ligand forming key interactions with residues Y111, R679, T678, V657, Y658, and C663. PCB 105 exhibits strong interactions with residues Y111, Y661, V657, Y658, and C663 of EZH2 (Figure 10B). PCB 118 forms multiple interactions with Y111, T678, R679, V657, Y658, and F665 (Figure 10C). The docking pose of PCB 138 reveals interactions with residues Y111, T678, R679, V657, Y658, and Y661 (Figure 10D). PCB 183 shows key interactions with residues Y111, E224, D223, T678, V657, Y658, Y661, and R679 (Figure 10E). PCB 187 interacts with an alternative set of residues, which includes S145, R64, Q66, Q273, Q276, and N143 (Figure 10F).

## 4 Discussion

**FIGURE 11:**
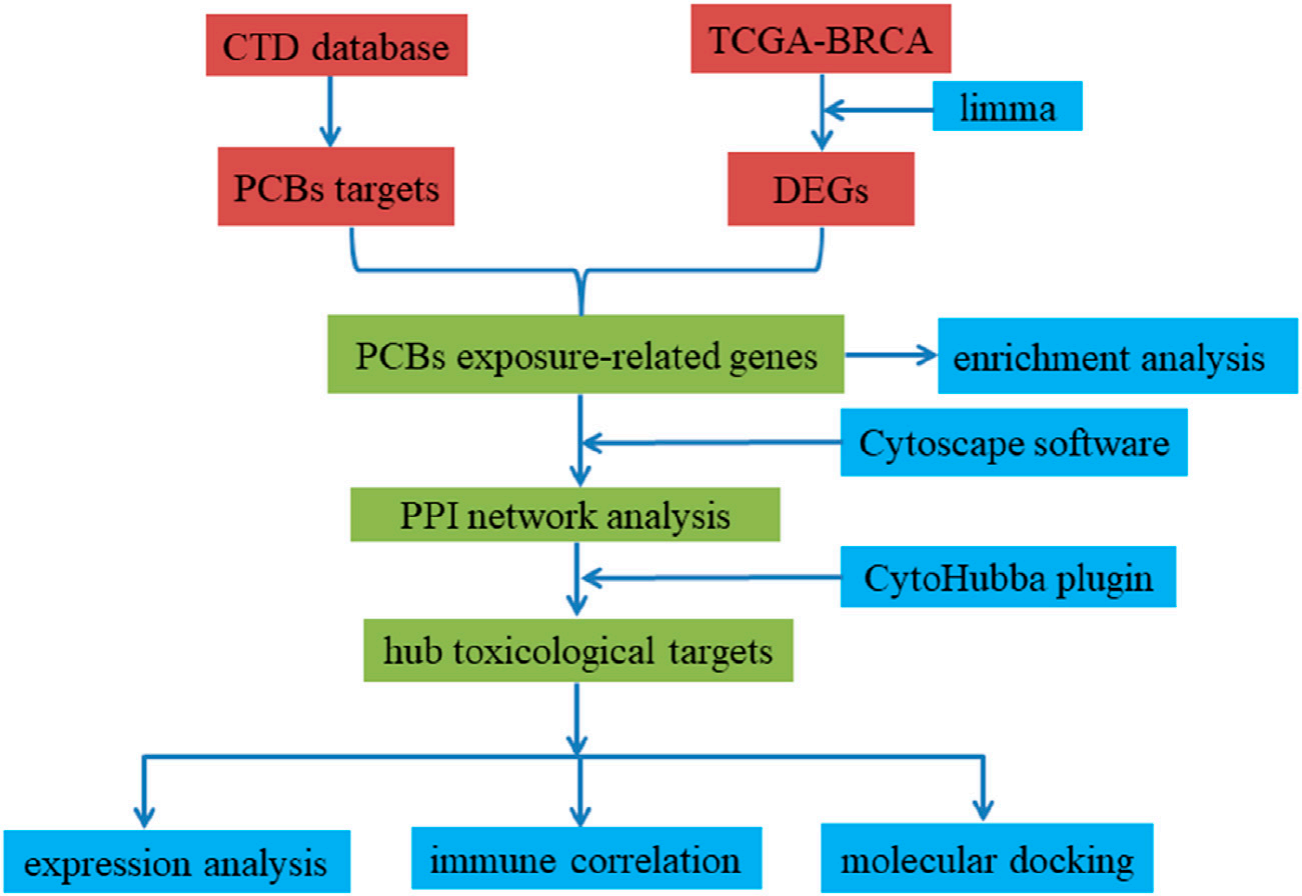
Methodology flowchart for this study.

Network toxicology represents an integrative discipline that synthesizes chemical informatics, systems biology, and bioinformatics, providing a robust framework for elucidating the mechanisms by which chemical agents can perturb biological molecular networks and compromise cellular homeostasis (Del Giudice et al., 2024). Molecular docking facilitates the simulation of complex chemical-protein interactions at the molecular level, thereby revealing potential mechanistic pathways through which these chemicals may influence the pathogenesis of cancer (Abd El-Meguid et al., 2022). Through the integration of these methodologies, this research aims to elucidate the molecular mechanisms by which chronic exposure to PCBs may modulate the initiation and progression of breast carcinoma. These findings offer a detailed molecular insight into the potential mutagenic, genotoxic, and carcinogenic effects of PCBs, linking identified targets to well-established toxicological endpoints such as DNA damage, genomic instability, and cellular transformation, which are critical in carcinogenesis. Figure 11 provides an overview of the research process. Our findings offer comprehensive insights into the molecular mechanisms by which PCBs promote breast cancer progression. By identifying key targets and pathways, this study provides potential biomarkers for breast cancer detection and highlights therapeutic intervention points. These findings are consistent with previous epidemiological and experimental evidence linking PCBs exposure to mutagenic and carcinogenic outcomes, as alterations in key signaling pathways such as PI3K-Akt and MAPK are recognized hallmarks of cancer progression (Parada et al., 2020; Wang D. et al., 2024). Understanding these mechanisms underscores the importance of addressing environmental pollutants in cancer research and aids in the formulation of public health policies to mitigate PCB exposure risks.

The strength of our study lies in the integration of network toxicology and molecular docking to systematically map PCB-induced perturbations in breast cancer-associated pathways. By combining differential expression analysis, enrichment analysis, and molecular docking, we identified several key toxicological targets and signaling pathways altered by PCBs in breast cancer, notably the PI3K-Akt and MAPK pathways, highlighting their essential roles in the disease’s pathology. These pathways are known to be involved in both genotoxicity and carcinogenicity, and their dysregulation by environmental pollutants like PCBs has been documented in several studies (Li et al., 2022; An et al., 2014; Radice et al., 2008; Song and Freedman, 2005). The PI3K-Akt pathway’s interaction with EZH2 and EGF is critical in this context. EZH2, as an epigenetic regulator, directly influences PI3K-Akt signaling by repressing tumor suppressor genes like PTEN (a key regulator of PI3K-Akt), thereby amplifying AKT1 activation and downstream survival signals (Yang et al., 2020; Wang J. et al., 2024). Similarly, EGF, via its receptor EGFR, activates both PI3K-Akt and MAPK pathways through phosphorylation cascades (Lv et al., 2015; Hwang et al., 2011). PCBs’ binding to EGF, as observed in our docking results, may stabilize EGFR-EGF interactions, prolonging MAPK/ERK and PI3K-Akt signaling, which drives proliferation and metabolic adaptation in tumor cells. This interaction aligns with the known mutagenic effects of PCBs, where persistent activation of growth signaling pathways can lead to DNA damage, mutation, and cellular transformation (Mutlu et al., 2016).

The PI3K-Akt pathway, known for its involvement in regulating cell survival, proliferation, and metabolism, is frequently dysregulated in breast cancer (Miricescu et al., 2020; Zhu et al., 2022; Sharma et al., 2017). Increased activation of this pathway, observed in our study through elevated AKT1 and BRCA1 expression, can lead to enhanced tumor cell survival and resistance to apoptosis (Foster et al., 2009; Wang et al., 2016). This dysregulation may serve as a potential target for preventive intervention, particularly given EZH2’s role in sustaining PI3K-Akt hyperactivity through epigenetic silencing of PTEN, as noted above. Such dysregulation of the PI3K-Akt pathway is a key contributor to PCB-induced carcinogenesis, as it has been shown in both animal and human studies that PCB exposure can lead to the genomic instability that fuels cancer progression (Ali et al., 2016; Qin et al., 2022). This aligns with previous research indicating that aberrant PI3K-Akt signaling contributes to aggressive breast cancer phenotypes and poor patient outcomes (Cancer Genome Atlas Network, 2012; Tian et al., 2021; Guo et al., 2023). In addition to the PI3K-Akt pathway, our study also highlights the significance of the MAPK signaling pathway in PCBs-induced breast cancer. The role of the MAPK pathway in cancer-related genotoxicity has been widely documented, with studies showing that PCBs exposure can promote mutagenic events through MAPK-driven proliferation and invasion (Zingales et al., 2021). This mechanistic link explains how PCB-EGF interactions could directly amplify oncogenic MAPK signalling, corroborating our observed disregulation of proliferation-associated genes. The MAPK pathway, which regulates cellular responses to growth signals and stress, also plays a significant role in cancer progression (Park and Baek, 2022; Santarpia et al., 2012). Our findings highlight that PCBs exposure leads to alterations in MAPK pathway components, such as AKT1 and EGF. Dysregulation of MAPK signaling has been associated with increased cell proliferation and metastatic potential in breast cancer (Wang et al., 2023; Wu et al., 2023). The upregulation of EGF, a key growth factor in this pathway, suggests that PCBs may promote tumor growth and progression by enhancing EGF receptor signaling, a mechanism previously linked to poor prognosis in breast cancer (Balanis and Carlin, 2017; Garcia et al., 2006). This underscores the interconnected roles of EZH2 and EGF in PCB-mediated pathway dysregulation: EZH2 epigenetically reinforces PI3K-Akt activation, while EGF-EGFR-PCB complexes drive parallel MAPK signaling, creating a synergistic oncogenic network. This dual pathway activation reinforces the carcinogenic potential of PCBs, as evidenced by their ability to induce genomic instability and mutagenic damage in breast cancer cells.

The recognition of EZH2, EGF, and AKT1 as pivotal toxicological targets in PCBs-mediated breast carcinogenesis provides critical insights into the molecular pathways through which PCBs facilitate tumor progression. EZH2, a histone methyltransferase and key epigenetic regulator, has been extensively linked to cancer progression through its role in transcriptional repression of tumor suppressor genes and promotion of cancer stem cell-like properties (Kim and Roberts, 2016). Overexpression of EZH2 is associated with poor prognosis in breast cancer (Pietersen et al., 2008). The involvement of EZH2 in PCB-induced carcinogenesis is supported by previous studies demonstrating that exposure to persistent environmental pollutants like PCBs can lead to epigenetic modifications, contributing to cancer risk (Singh, 2024). Our molecular docking analysis, which demonstrated strong binding between PCBs and EZH2, hypothesizes that PCBs exposure could potentially influence EZH2’s oncogenic activity, possibly leading to epigenetic modifications that promote tumor growth and therapy resistance. This aligns with epidemiological studies indicating that individuals exposed to high PCB levels have increased risks of developing cancers with epigenetic alterations (Guo et al., 2024). Targeting EZH2 may represent a potential preventive strategy for high-risk populations exposed to PCBs, offering a molecular approach to mitigate pollutant-induced epigenetic alterations. EGF is a well-known growth factor that drives tumor cell proliferation and survival through its activation of the EGFR signaling pathway (Feng et al., 2012; Lee et al., 2021). Similarly, the strong interaction between PCBs and EGF underscores the significance of EGF in breast cancer pathology.

However, it is important to acknowledge the limitations of our study. While the molecular docking results and pathway analysis provide valuable insights into the potential mechanisms by which PCBs influence breast cancer progression, we cannot draw definitive conclusions about the clinical applicability of these findings. The relationship between PCBs exposure and breast cancer is based on epidemiological data, and it remains challenging to identify individual patients whose breast cancer may be directly attributed to PCBs exposure. Further clinical and molecular studies are needed to validate the toxicological relevance of these findings in the context of human breast cancer. Thus, while the targets identified in our study are relevant for understanding the biological mechanisms involved, further research, including clinical validation, is required before these findings can inform therapeutic strategies. Functional assays, such as gene knockdown experiments, would be necessary to confirm the mechanisms suggested in this study and provide direct evidence of the molecular interactions proposed. Additionally, rather than speculating about individualized therapies, future studies should aim to better characterize the epidemiological and molecular associations between PCBs exposure and breast cancer. It is also crucial to explore the combined effects of PCBs exposure with other environmental pollutants, including metals, dioxins, and chlorinated pesticides, which may synergize to exacerbate carcinogenic outcomes. One potential direction is to explore the role of environmental pollutants in contributing to cancer-related health disparities and whether certain populations are more vulnerable to PCBs-induced carcinogenesis. Considering the environmental and biological complexities of PCBs exposure, especially as a mixture of chemicals, further studies should address how cumulative exposure and interactions with other pollutants affect breast cancer risk. Further research into the molecular signatures of PCBs-exposed tumors may also provide opportunities to develop broader preventive strategies targeting the identified pathways, including the PI3K-Akt and MAPK signaling pathways. These findings could contribute to public health measures aimed at reducing PCBs exposure and preventing breast cancer.

## 5 Conclusion

In conclusion, our study underscores the importance of addressing environmental pollutants such as PCBs in cancer research. By identifying key molecular targets and pathways involved in PCB-induced breast cancer, we provide a foundation for future studies aimed at developing targeted therapies and preventive measures. The integration of network toxicology and molecular docking represents a powerful approach to elucidate the complex molecular interactions underlying environmental pollutant-induced cancers, and our findings highlight the potential of this approach to uncover novel therapeutic intervention points.

## Data Availability

The original contributions presented in the study are included in the article/Supplementary Material, further inquiries can be directed to the corresponding authors.
