# Supplementary material for: Molecular mechanisms of polychlorinated biphenyls in breast cancer: insights from network toxicology and molecular docking approaches
Source: Front Pharmacol. 2025 Jun 13;16:1604993. doi: 10.3389/fphar.2025.1604993 (PMC12202659; doi:10.3389/fphar.2025.1604993)
Supplement: Supplementary file 1 [file Supplementaryfile1.docx]

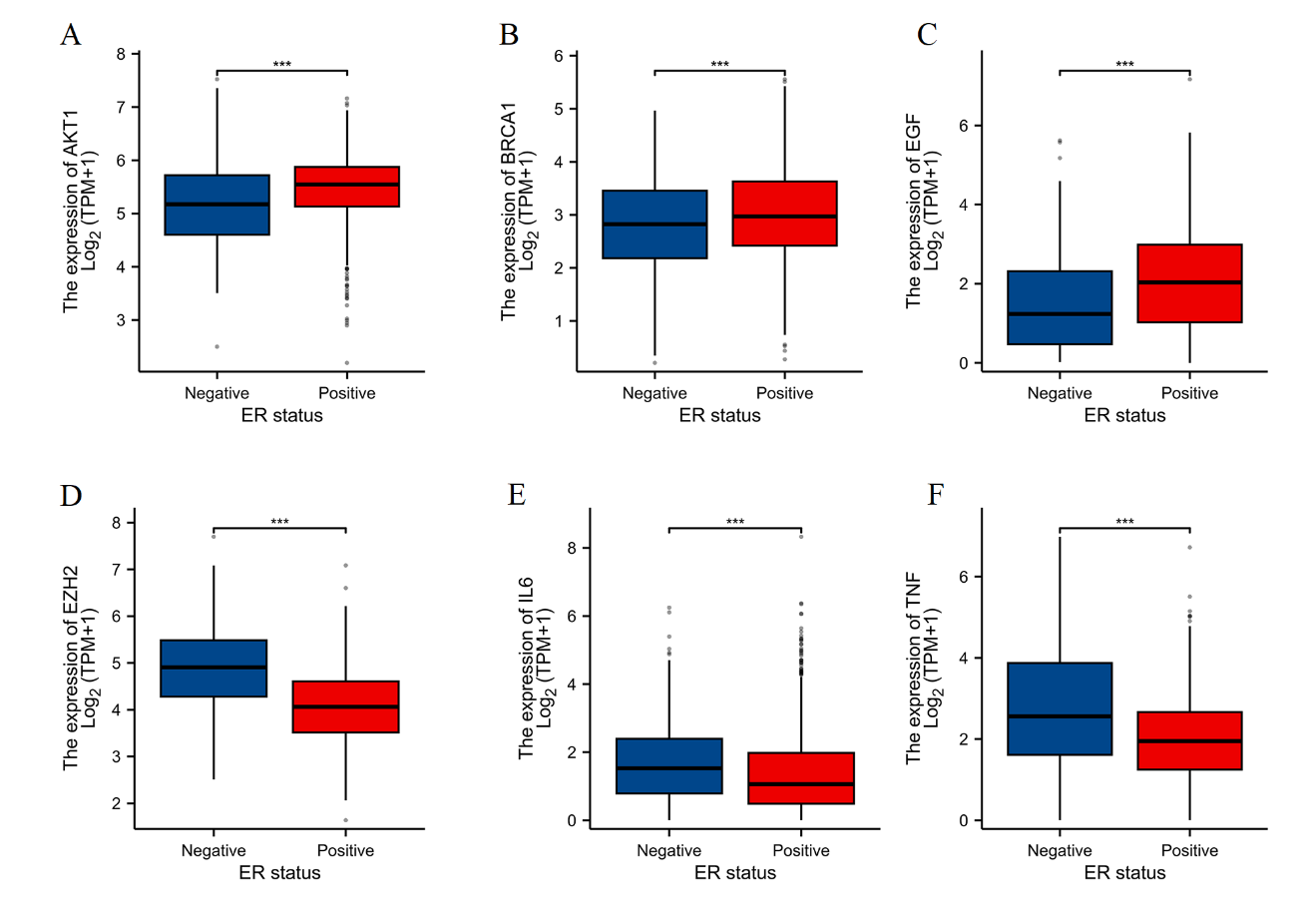


Figure S1 Differential expression of key PCBs-related toxicity targets in breast cancer subtypes based on ER status. Expression levels (Log2(TPM+1)) of six key PCBs-related toxicity targets were analyzed in the TCGA-BRCA dataset: (A) AKT1, (B) BRCA1, (C) EGF, (D) EZH2, (E) IL6, and (F) TNF. Each boxplot compares expression levels between ER-negative (blue) and ER-positive (red) breast cancer samples.


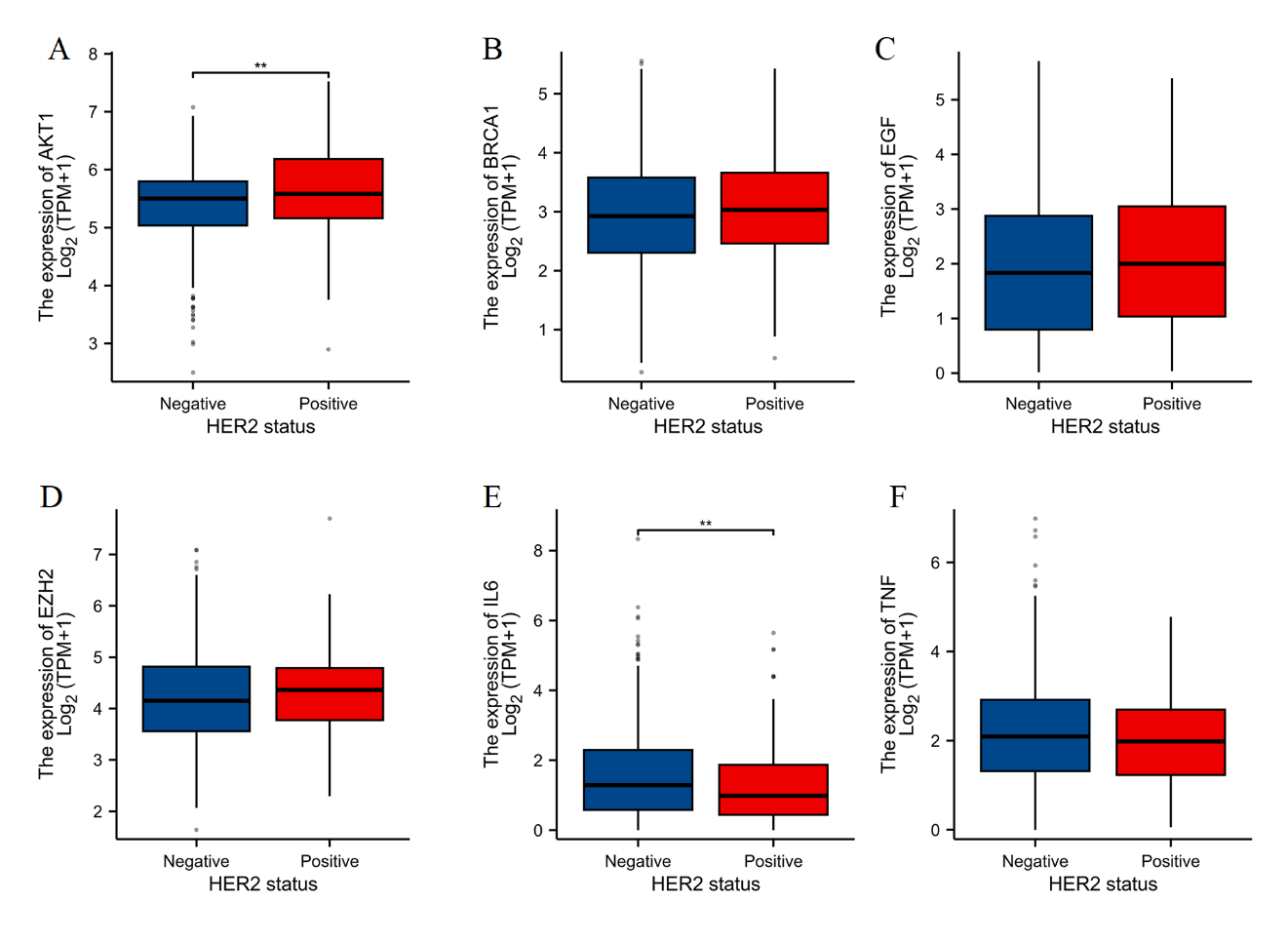


Figure S2 Differential expression of key PCBs-related toxicity targets in breast cancer subtypes based on HER2 status. Expression levels (Log2(TPM+1)) of six key PCBs-related toxicity targets were analyzed in the TCGA-BRCA dataset: (A) AKT1, (B) BRCA1, (C) EGF, (D) EZH2, (E) IL6, and (F) TNF. Each boxplot compares expression levels between HER2-negative (blue) and HER2-positive (red) breast cancer samples.


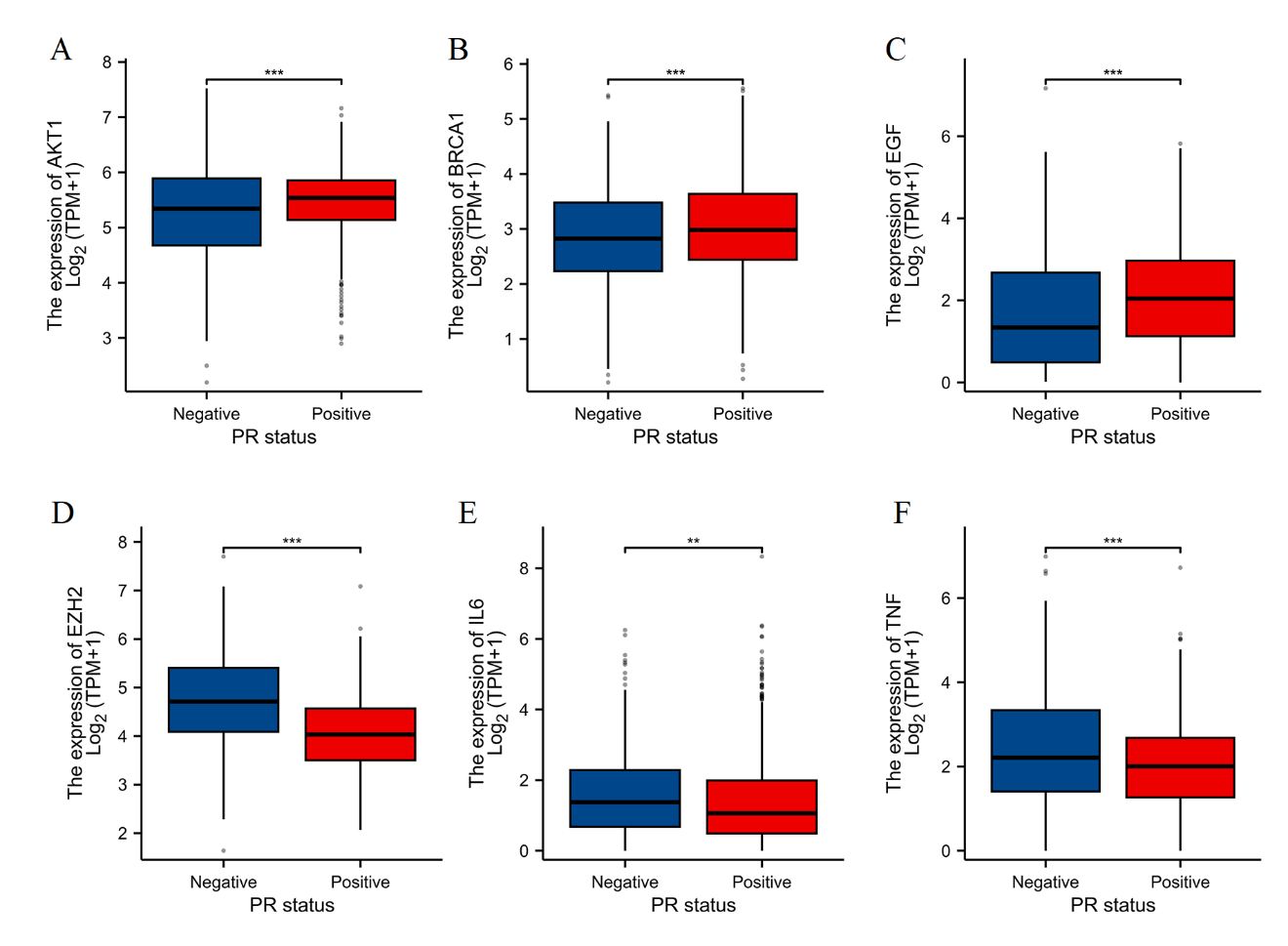


Figure S3 Differential expression of key PCBs-related toxicity targets in breast cancer subtypes based on PR status. Expression levels (Log2(TPM+1)) of six key PCBs-related toxicity targets were analyzed in the TCGA-BRCA dataset: (A) AKT1, (B) BRCA1, (C) EGF, (D) EZH2, (E) IL6, and (F) TNF. Each boxplot compares expression levels between PR-negative (blue) and PR-positive (red) breast cancer samples.
